# Mind the Gap – Ideas for Making Clinical Research More Relevant for Practitioners and Patients

**DOI:** 10.32872/cpe.12419

**Published:** 2024-03-28

**Authors:** Max Berg, Lea Schemer, Lukas Kirchner, Saskia Scholten

**Affiliations:** 1 Clinical Psychology Group, University of Marburg, Marburg, Germany; 2 Department of Clinical Psychology and Psychotherapy, RPTU Kaiserslautern-Landau, Landau, Germany; Philipps-University of Marburg, Marburg, Germany

Randomized controlled trials (RCTs) are widely considered to be the gold standard for demonstrating *efficacy* in psychotherapy research. However, the *clinical utility* of “typical” RCTs for establishing routine care therapies has been a topic of long-standing debate in our field (Persons & Silberschatz, 1998). “Typical” refers to a study with a small to moderate sample size that targets a disorder according to a standardized diagnostic manual and is often waiting-list controlled (Carey & Stiles, 2016). Practitioners frequently express criticism about the external validity of such RCTs (Gyani et al., 2015; Safran et al., 2011). In qualitative investigations, therapists describe the “unrepresentativeness of RCTs” as a reason for why they do not regard clinical research as an important foundation for their everyday decision making (Gyani et al., 2015). A review suggested that the perceived “inflexibility” of manuals could also be related to the lack of interest of many practitioners and that therapists wonder whether the “standardized instructions” provided in them are useful for their heterogeneous clinical use cases (Speers et al., 2022).

Similarly, from a methodological point of view, the inference to intra-individual variability from group-level research was challenged (Fisher et al., 2018). Furthermore, the substantial heterogeneity in treatment effects suggests that even if patients with the same diagnoses are treated with the same treatment by the same therapist, they respond differently (Herzog & Kaiser, 2022). Given the methodological challenges and the skepticism of therapists, we argue that the criticism regarding clinical science should be taken seriously. In this editorial, we present five ideas for improving psychotherapy research and for addressing the research practice gap.

## Five Ideas for Psychotherapy Research

### Idea One: Focus on Transdiagnostic Mechanisms

Diagnoses in clinical psychology typically do not present homogeneous entities, and comorbidity rates between “different” disorders are commonly high (Rief et al., 2023). For example, different patients exhibit largely heterogeneous symptom dynamics in depression and novel clinical research is starting to acknowledge this (Fried et al., 2023). Also, we know from a large body of research that pathological mechanisms are not limited to a single disorder, but oftentimes pose transdiagnostic problems (Dalgleish et al., 2020). Transdiagnostic mechanisms include (but are not limited to), dysfunctional expectations with aberrant belief updating (Kirchner et al., 2022), social impairments (Lehmann et al., 2019), and reward insensitivity and its interplay with stress dysregulation (Martin-Soelch, 2023). Given the potential of transdiagnostic mechanisms, it seems worthwhile to allocate treatment based on them rather than solely based on diagnoses. For example, patients who exhibit a high tendency for repetitive negative thinking could be assigned to focused therapies that target this mechanism, regardless of whether they have been diagnosed with depression, generalized anxiety disorder, or both.

### Idea Two: Dismantle Treatment Protocols

A plethora of therapeutic techniques exist to target (transdiagnostic) mechanisms (Schaeuffele et al., 2021). Yet, we know little about their isolated effect because treatment manuals oftentimes with overlapping strategies – are evaluated as a *treatment package*. Such “evidence-based black boxes” are effective for treating numerous mental disorders, but their respective effect sizes and response rates remain moderate (Ormel et al., 2022). Future research should dismantle treatment protocols and evaluate the effect of specific techniques. Applying dismantled techniques instead of treatment protocols might be closer to clinical practice anyway, where the implementation of complex procedures is limited due to time, comorbidity patterns, and financial resources. The dismantling of treatment packages may also necessitate a departure from traditional therapy orientations. Competence-oriented frameworks (Rief, 2021), or process-based therapy (Moskow et al., 2023) are two approaches that could promote a more “toolbox oriented” thinking.

### Idea Three: Monitor Individual Trajectories With Sufficient Resolution

In clinical research and practice, diagnostic instruments are usually collected at only a few points in time (e.g., before and after treatment). To date, few projects exist that collect intensive longitudinal data (i.e., session-by-session data or ecological momentary assessment) in clinical trials and routine care settings (Lutz et al., 2022). These methods would allow to monitor individual trajectories, compare patients to similar cases and provide computerized treatment suggestions, while reducing therapist biases regarding outcome estimation (Lutz et al., 2022). M-Path and Shiny apps are digital implementations of such efforts (Mestdagh et al., 2023). However, just because appropriate tools are available does not mean they are already being frequently used. Barriers, particularly in terms of usability and knowledge of digital technologies, can make it difficult for clinicians to use digital innovations. Therefore, it is vital that the curricula of psychology students are expanded from science- to practice-oriented use of data literacy and computer science.

### Idea Four: Use Causal Inference Methods for Routine Care Data

Large psychopathology data sets exist in routine care, but we need to sample and process them in a way that allows for causal inference. It was suggested that we can develop alternatives to RCTs for estimating the causal effect of a given treatment on an outcome. In addition to established approaches like propensity score matching (Lee & Little, 2017), single-case experimental designs can be utilized as an ideographic alternative for RCTs. These designs utilize an experimental manipulation that compares the individual response of a patient at different time points. (e.g., during treatment delivery versus waiting-periods). Single-case experimental designs have the potential to empower practitioners to become scientist-practitioners of their own clinical practice (Kazdin, 2019).

“Synthetic waitlists” can also bring causal inference into psychotherapy research (Kaiser et al., 2023). Here, machine learning algorithms select patients from waiting lists, based on the multidimensional similarity to a given patient under treatment. This, in turn, allows to estimate the probability that a specific patient would have reached a certain outcome without receiving therapy. If this probability is low, then a significant part of the improvement can be attributed to the treatment. Utilizing synthetic waitlists allow us to harvest routine data as an additional source of information and estimate the effect of therapeutic strategies under realistic, everyday conditions.

### Idea Five: Utilize the Expertise of Practitioners and the Lived Experience of Patients

Participatory science actively engages various stakeholders throughout the entire research process (Slattery et al., 2020). For example, patients should be involved as experts in the development of clinically relevant research questions (Birnie et al., 2019), the optimization of treatment manuals (Schemer et al., 2023), and for the planning of upcoming research projects (Slattery et al., 2020). Similarly, practitioners can be involved to facilitate the clinical usefulness of technological advances or to find ways to overcome practical barriers to implement an effective therapeutic strategy. Here, we face the challenge of involving practitioners who are distant or even skeptical about clinical research. Yet, a participatory approach would help to address the research-practice gap by involving groups for whom practically relevant and effective clinical science is in their vital self-interest.

## Conclusion

In conclusion, addressing the research-practice gap requires a shift towards dismantling the effect of specific therapeutic techniques on better-operationalized transdiagnostic mechanisms. Monitoring individual trajectories and using innovative methods for inference can provide valuable insights into therapy effectiveness, if needed at an individual level. Finally, an active involvement of non-scientists can create research that is interesting and engaging for different stakeholders.
